# Evidence for a stable single component sharp texture in high purity aluminum during tube high-pressure shearing at room temperature

**DOI:** 10.1038/s41598-022-21717-z

**Published:** 2022-10-25

**Authors:** Zheng Li, Luo Yi Li, Ye Bin Zhu, Kui Lin, Zhi Tian Ren, Yang Yang, Ying Liu, Jing Tao Wang, Terence G. Langdon

**Affiliations:** 1 School of Materials Science and Engineering, Nanjing University of Science and Technology, Nanjing, 210014 China; 2 Materials Research Group, Department of Mechanical Engineering, University of Southampton, Southampton, SO17 1BJ UK

**Keywords:** Metals and alloys, Design, synthesis and processing

## Abstract

A stable {$\stackrel{\mathrm{-}}{1}{\text{10}}$}  <110>  single component sharp texture was obtained during ambient temperature *tube* High-Pressure Shearing (*t*-HPS) of 99.999% purity aluminum. It is shown that the grain size and the grain aspect ratio saturate at ~ 8 μm and ~ 1.6, respectively, at an equivalent strain of ~ 30 and the high-angle grain boundary fraction continues to decrease after this saturation even to equivalent strains exceeding ~ 200. The {$\stackrel{\mathrm{-}}{1}{\text{10}}$}  <110> texture emerges at an equivalent strain of ~ 6 to 9 with the completion of recrystallization and develops gradually as a sole component sharp texture with increasing intensity upon further processing. This component is a stable orientation in *t*-HPS processing although it was not previously observed experimentally as a shear texture. Thus, *t*-HPS processing provides a new and effective experimental tool for simple shear testing that is distinctly different from earlier shear strain methods such as torsional processing.

## Introduction

In terms of texture control, it is important to consider the cube texture {001} <100>. This is a well-known example of the development of prominent crystallographic texture via the process of deformation followed by recrystallization in metallic materials, which was first observed more than 90 years ago in a copper sheet with heavy rolling and subsequent annealing^1^. Subsequently, this texture was shown to develop in different face-centered cubic (fcc) metals (e.g., Cu, Ni and Al) and alloys, by heavy cold rolling and recrystallization annealing^2–5^ or hot rolling^6^.

This cube texture is observed to be weakened by the surface shear from friction in the rolling before recrystallization^7–9^. This raises the question of how would the texture develop under complete shear strain. It is well known that there are two different types of strain which are designated normal strain and shear strain^10^. In practice, normal strain dominates deformation such as rolling and leads to the sharp cube recrystallization texture in fcc metals. A parallel understanding of the texture evolution during and after shear strain dominated deformation would complete the knowledge of the effect of all types of strain on the recrystallized texture.

In terms of shear, the simplest way to directly apply a shear strain to materials is by a shear test but the strain level is then limited by the overall sample dimensions. Alternatively, torsion has been utilized as a standard test to characterize material performance in shear for over two centuries^11,12^ and also it is widely used to characterize the microstructural evolution of materials in simple shear processed to high strain levels^13,14^. High-pressure torsion (HPT) processing is a conventional severe plastic deformation (SPD) procedure which further improves the mechanical stability of the torsion process by applying a hydrostatic pressure so that, theoretically, an unlimited shear strain may be achieved^15,16^. It has been confirmed in many investigations that these shear-dominated processes produce highly comparable shear textures^17–19^. However, there is no report of the development of a single component texture, either in the as-deformed state or in recrystallization following torsion or HPT of fcc metals or alloys alloys^20^.

For the previously mentioned strong cube texture development after annealing, heavy cold reduction in unilateral rolling is required^21^ and a strain path change, such as cross-rolling, was reported to be critically adverse for the development of an annealing cube texture subsequent to rolling^21^. This suggests that the reported diversity in the texture development in annealing/recrystallization during or after torsion or HPT processing may be attributed to the underdevelopment of texture from path changes during the processing.

As a confirmation of the above perception, torsion textures are reported rarely strong^22^. It is also anticipated there are no stable orientations because any particular grain is constantly rotating and the presence of a texture is simply the result of the presence of quasi-stationary positions in the orientation distributions where grains rotate very slowly with respect to the specimen axes^23^. Furthermore, the high axial pressure in HPT leads to axial compression^24^ and obvious and complex sample extensions or contractions along the sample axis^25^ are frequently observed in torsion experiments. These factors, similar to the cross-rolling passes in rolling^21^, may also lead to the underdevelopment of a deformation texture and this in turn influences the development of a strong single component texture in any subsequent annealing. An example of the underdeveloped texture during such torsional processes is the weakening of the major torsion texture after passing through an intensity peak upon continuous single direction torsion^26^. It is therefore apparent that, although torsion may produce much higher strain levels than rolling, the torsion texture appears to be relatively weaker by contrast to the heavy rolling texture^27^.

**Figure 1:**
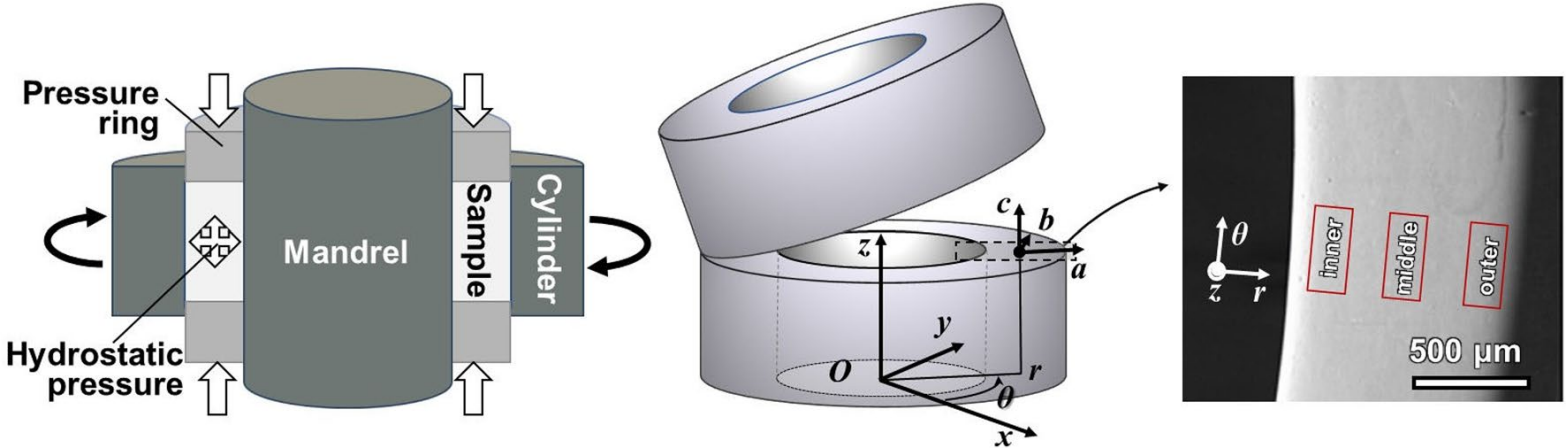
Illustration of the principle of *t*-HPS (left), the sample tube for *t*-HPS in cylindrical coordinates (center), half-height annular section of the tube wall with the observation regions: inner, middle and outer (right). Two sets of coordinate systems are defined: the macroscopic sample coordinate system is expressed in cylindrical coordinates ***r–θ–z*** and the local Cartesian coordinate system in terms of ***a–b–c***. At any local position, these two coordinate systems always keep ***r*** parallel to ***a***, ***θ*** parallel to ***b***, and ***z*** parallel to ***c***.

An examination shows that the common feature of the factors that lead to an underdevelopment of the deformation texture, such as in cross-rolling, axial compression in HPT and axial extensions or contractions in torsion, and all lead to a breaking of the plane strain condition. Therefore, an unchanged shear direction and a plane strain requirement throughout the deformation process, or simple shear^28^ to a high strain level, appear to be critical for the development of a strong deformation texture. For this reason, a new simple shear process other than torsion or HPT is required and this may be achieved by using the new deformation process of *tube* High-Pressure Shearing (*t*-HPS)^29^. This alternative shear technique was chronologically first developed as rotation shear (RS)^30^ and thereafter there were different subsequent versions of high pressure tube twisting (HPTT)^31,32^ and *t*-HPS. According to earlier analyses^29,33,34^, processing by *t*-HPS, as shown schematically in left of Fig. 1, which produces a plane shear strain with a constant shear direction (azimuthal), appears to be a potential processing technology to develop a strong deformation texture that may induce a single component texture in appropriate subsequent treatments.

The present research was therefore motivated by these considerations. Accordingly, a large shear strain was realized in single path *t*-HPS and the microstructural and texture evolution was characterized by electron back-scatter diffraction (EBSD). In order to simplify the investigation, high purity 5 N (99.999wt%) aluminum with a high SFE was selected as a model material to avoid the effect of impurities and any additional complexities in the deformation mechanisms on the microstructure/texture evolution. It is worth mentioning that microstructural and texture control are also important requirements for the application of high purity aluminum in modern technology as in metallizing in the integrated circuit engineering.

## Results

In order to clearly display the results, a global cylindrical coordinate system ***r–θ–z*** was established in the *t*-HPS process together with a local Cartesian coordinate system ***a–b–c*** which is used to indicate the observation sections, as shown in Fig. 1. Representative samples were taken from the annular cross-sections at half height of the tube to avoid the complexity of edge effects^29,34^.

### The microstructure at a pressurized stage before *t*-HPS rotation

**Figure 2:**
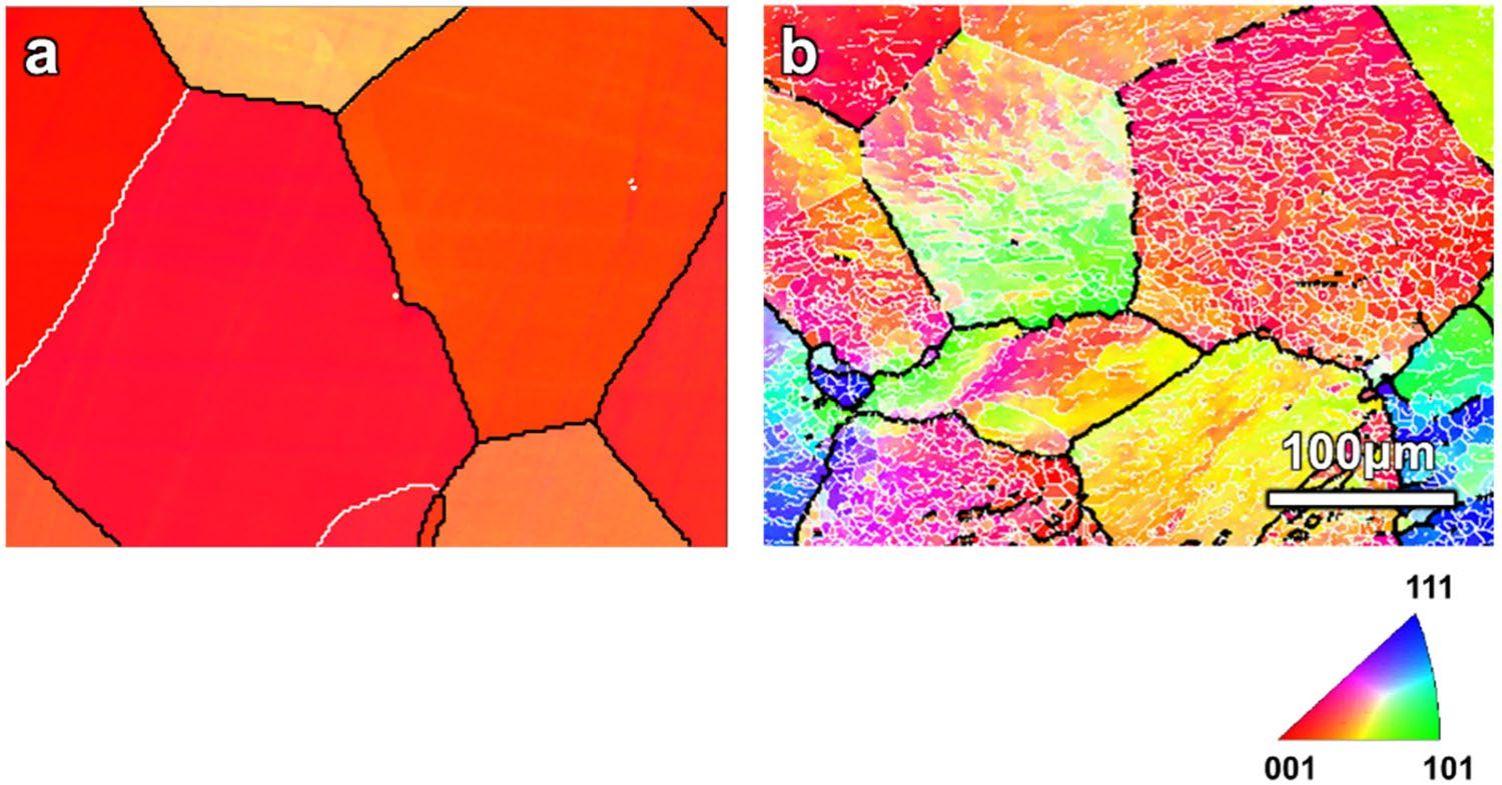
The Inverse pole figure (IPF) map obtained by EBSD showing (**a**) the fully-annealed coarse grains of 5N Al before *t*-HPS and (**b**) the sub-grain boundaries (white nets) formed in these coarse grains when the sample is subjected to a hydrostatic pressure of ~ 3 GPa. The scale bar is the same in (**a,b**).

The average grain size in the as-received and annealed sample before *t*-HPS was ~ 250 μm based on statistics from more than 500 grains in scanning electron microscopy (SEM). Figure 2a gives a typical example of this coarse-grained microstructure of equiaxed grains with straight and sharp boundaries which is typical of a fully-annealed equilibrium grain structure.

Figure 2b shows the microstructure of the 5 N Al after the pressure reaches ~ 3 GPa before any rotation. All grains remain equiaxed with straight high-angle grain boundaries but there now emerges some low-angle boundaries within the grains. This indicates that a small plastic strain was introduced at this pressurization stage and this led to low-angle boundaries where the misorientations were not sufficient to cause any obvious distortions of the original high-angle grain boundaries. Strong dynamic recovery is also indicated by the sharp subgrain boundaries. Because of the strong dynamic recovery due to the high SFE of aluminum, residual dislocations rearrange into various low-angle dislocation interfaces such as dislocation cell walls (misorientations ~ 1°), high density dislocation walls (misorientations < 2°) or low-angle grain boundaries (misorientations < 10°–15°)^35^. In Fig. 2b, considering the spatial and angular resolutions of the EBSD technique, the interlacing meshes at the micron scale within the coarse grains are sub-grain boundaries with misorientations larger than 2°.

### Dynamic recrystallization during *t*-HPS

#### Partial recrystallization

**Figure 3:**
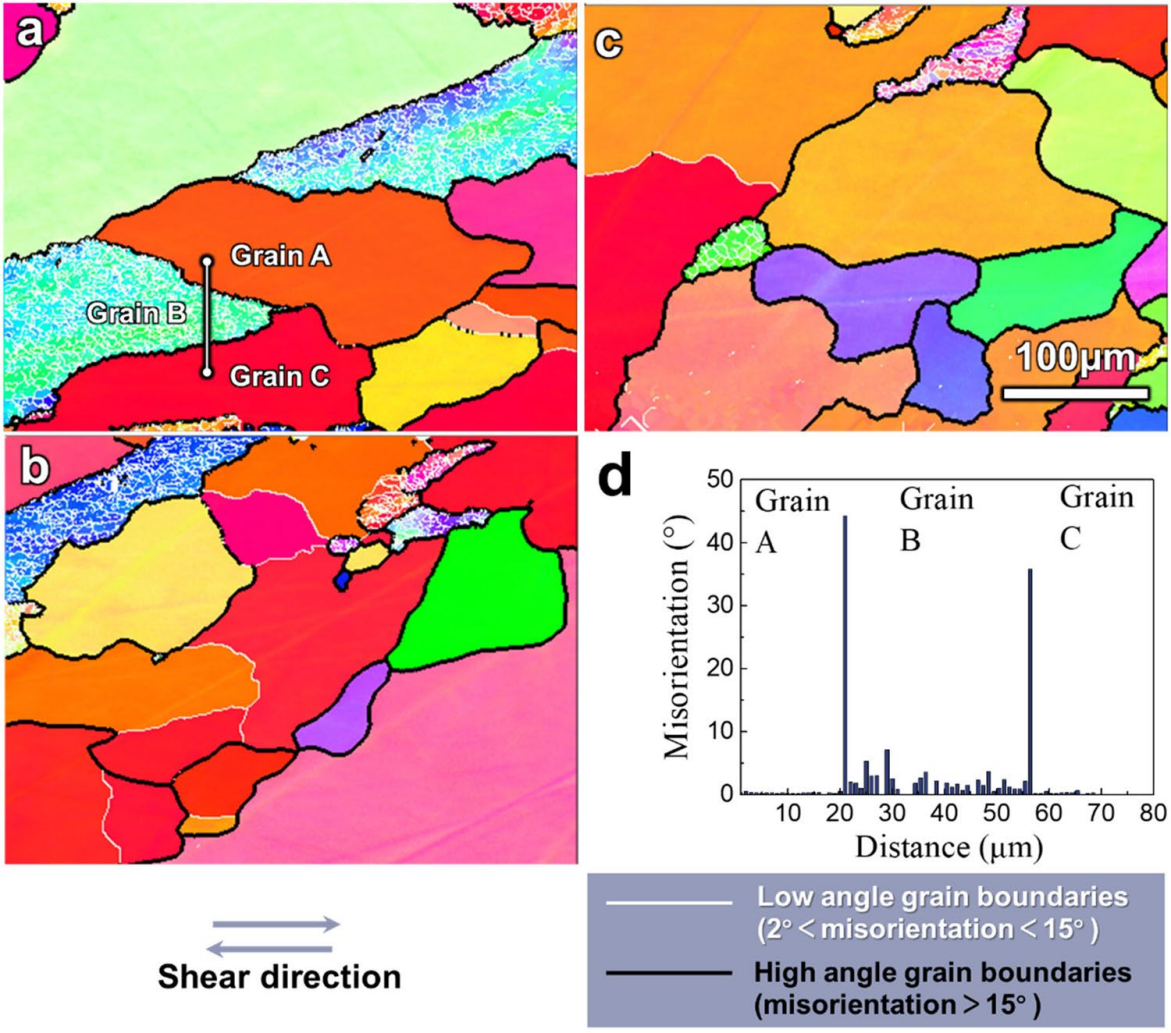
EBSD IPF maps from inner (**a**), middle (**b**) and outer (**c**) regions of the 5N Al processed by *t*-HPS to a rotation angle of π/6, and (**d**) the misorientation changes from grain A to C across B as shown in (**a**). The scale bar is the same in (**a–c**).

Figure 3 presents the microstructure as EBSD IPF maps of the sample with a *t*-HPS rotation of *π*/6. The high-angle grain boundaries (HAGBs) are no longer straight but seriously distorted after *t*-HPS processing and this contrasts with the as-annealed and pressurized samples. The spacing between the HAGBs along the shear orientation (horizontal) is larger than along the radius (vertical). Figure 3d gives the misorientation change along the line segment across three grains labeled A, B and C in Fig. 3a. The neighboring grains A/B or B/C are separated by HAGBs and within grains A and C there is no visible misorientation but only some limited noise. By contrast, in grain B there are clearly defined low-angle grain boundaries (LAGBs) with misorientations less than 15° and sub-grain size ranges from 1 to 2 μm. The re-emergence of clean grains that are free of subgrain boundaries, by contrast with the microstructure at the pressurization stage, indicates complete recrystallization in these grains whereas in the un-recrystallized areas the recovery maintains a sub-grain structure.

#### The completion of recrystallization and the saturation of grain refinement

**Figure 4:**
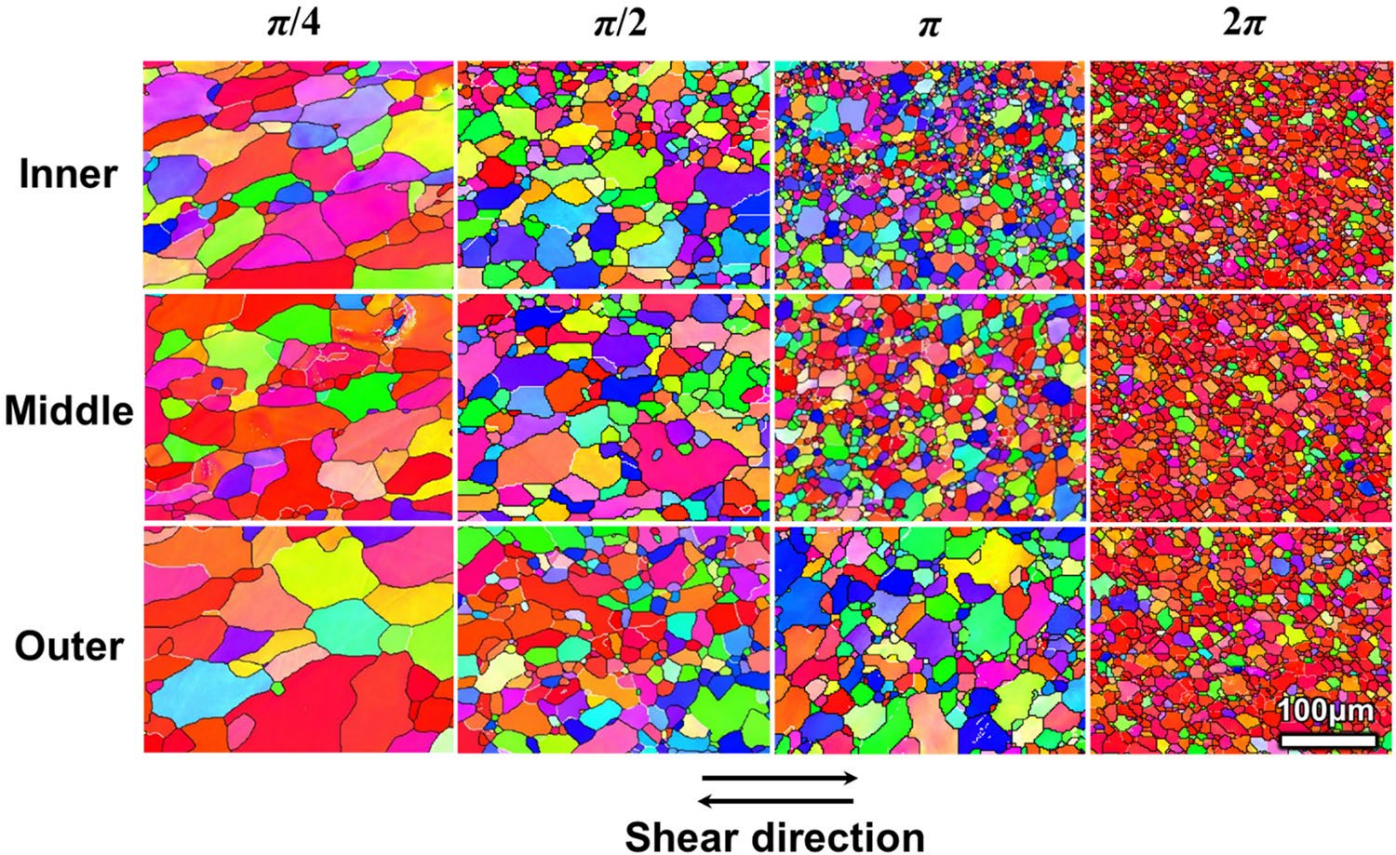
EBSD IPF maps from inner (top), middle (center) and outer (bottom) regions as indicated in Fig. 1 of the 5N Al processed by *t*-HPS to a rotation angle of π/4 (left), π/2 (center left), π (center right) and 2π (right). The scale bar is the same for all the IPF maps.

Figure 4 gives the microstructures as EBSD IPF maps for samples with *t*-HPS rotations of *π*/4, *π*/2, *π* and 2*π*. No sub-grain structure is observed when the rotation angle reaches *π*/4 and above, thereby indicating a completion of dynamic recrystallization during *t*-HPS. This is reasonable since dynamic recrystallization of large-strain compressed aluminum was reported at ambient temperature^36^.

It is evident from Fig. 4 that the grains are refined as the rotation angle of *t*-HPS further increases. Due to the radial strain gradient of *t*-HPS, the grains near the inner surface are always smaller than near the outer surface at the same rotation angle. Fig. S1 in the supplementary materials summarizes the grain size change from the inner surface to the outer surface at different rotations of *t*-HPS and these data show the grain refinement and grain size gradient upon *t*-HPS. An average grain size of 8 ± 5 μm was obtained near the inner surface of the tubular samples at half rotation. The radial grain size gradient diminishes gradually with increasing *t*-HPS rotation angle and thereafter it becomes difficult to recognize such a gradient when the *t*-HPS rotation angle reaches 2*π* or a full rotation where the average grain size saturates at 8 ± 5 μm. The grain aspect ratio also decreases and saturates, accompanying the grain refinement, to a value of ~ 1.6. This is consistent with the results of dynamic recrystallization at large strains in SPD processes which lead to nearly equiaxed refined grains^37^.

#### The evolution of grain boundary misorientations

**Figure 5:**
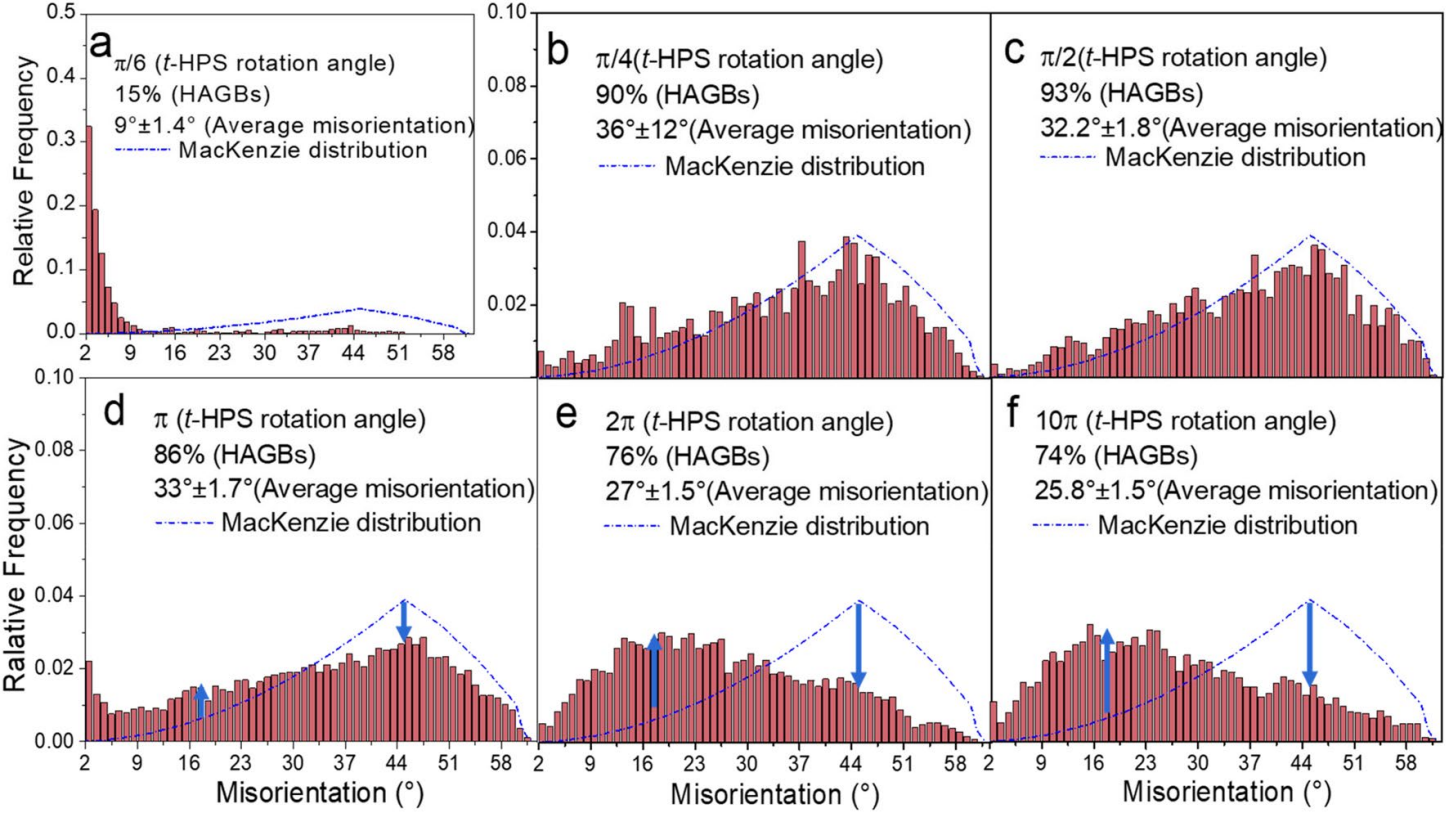
Evolution of grain boundary misorientation distributions upon increase of *t*-HPS rotation angle through (**a**) π/6, (**b**) π/4, (**c**) π/2, (**d**) π, (**e**) 2π and (**f**) 10π. It is important to note the different range of the vertical axis in (**a**).

The distribution of misorientations between neighboring grains is shown in Fig. 5. Except for the *t*-HPS rotation of *π*/6 where the HAGB fraction is ~ 15%, for all *t-*HPS rotations at or above *π*/4 the microstructures are observed with complete recrystallization. In addition, the HAGB fraction is above ~ 75% and even to 93% for a rotation of *π*/2 which is reasonably close to the fully recrystallized structure with random orientation of ~ 97%^38,39^. By comparison, a typical value of the HAGB fraction for microstructures saturated in SPD is about 70 ~ 80%^40–42^. There is no evidence for the retention of special boundaries in these diagrams.

**Figure 6:**
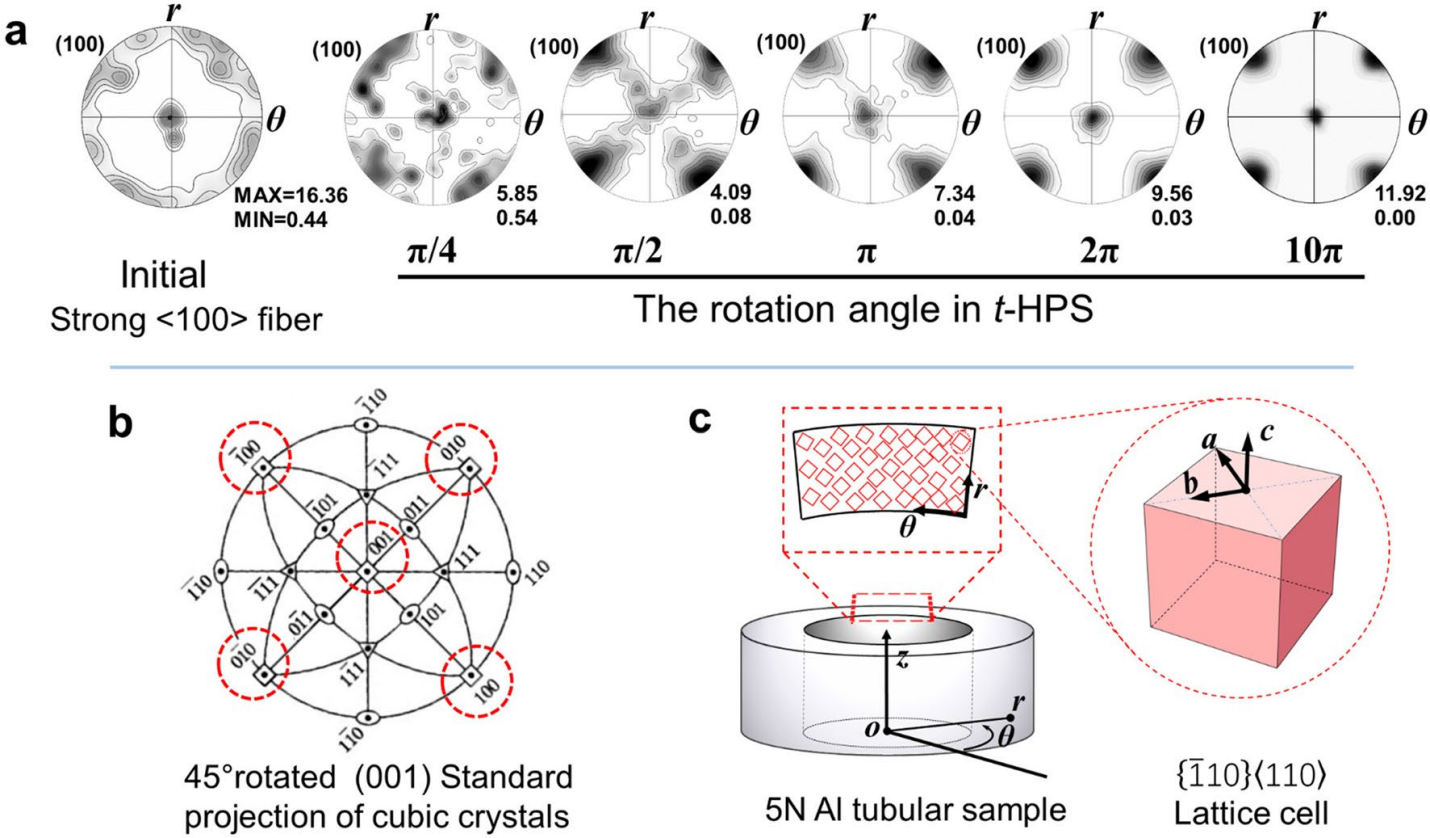
(**a**) (100) pole figures of the as-received 5N Al sample bar and after *t*-HPS processing; (**b**) 45° rotated (001) standard projection of cubic crystal and (**c**) the illustration of an ideal {$\stackrel{\mathrm{-}}{1}{\text{10}}$}  <110> lattice cell with its relation to the *t*-HPS tube sample.

A typical feature of these plots in Fig. 5 is the evolution of the misorientation distributions so that they fit the Mackenzie distribution of random misorientations^38,39^ for *t*-HPS rotations of *π*/4 and *π*/2 with a peak at 40°–50°. This peak drops significantly at a *t*-HPS rotation of *π* and almost disappears at a *t*-HPS rotation of 2*π* where it is substituted by a new peak at a lower misorientation angle of 15°–25°. This tendency of deviation of grain boundary misorientation from the Mackenzie distribution demonstrates that the orientation differences between grains are decreasing, where this implies a continuous enhancement of deformation texture as confirmed by the results to be presented in Fig. 6.

### The texture evolution

The (100) pole figures of the as-received 5 N Al sample is shown in Fig. 6a, with specific texture components along the <100> fiber resulting from extrusion for the production of the material.

After *t*-HPS processing, a new texture component gradually emerges, then strengthens and finally becomes the only texture component at a *t*-HPS rotation angle of *π*, as shown in Fig. 6a. According to the standard stereographic projection in Fig. 6b, the ideal orientation of this single-component texture is {$\stackrel{\mathrm{-}}{1}{\text{10}}$} <110> by the Miller indices, where the normal of the {$\stackrel{\mathrm{-}}{1}{\text{1}}{0}$} crystallographic plane is parallel to the radius ***r*** and the  <110> crystallographic direction is parallel to the azimuthal direction ***θ*** of the tube. This leads to the < 001 > crystallographic direction parallel to the tube axis ***z*** as illustrated in Fig. 6c.

To compare the single component texture with those of the well-established ideal orientations of simple shear fcc metals as presented in Table S1^19^, the (111) pole figure of the sample with a *t*-HPS rotation angle of *π* was overlapped on the ideal orientations of the shear texture of fcc metals, as in Fig. S2, where the ideal orientation of the single component texture observed in this work is marked by yellow stars. It is obvious that this single component texture is different from the deformation textures usually found for simple shear fcc metals^19^.

The relative texture intensity obtained from the (100) pole figure (red squares) and the (111) pole figure (blue stars in the insert), upon an increase of the *t*-HPS equivalent strain, are shown in Fig. S3. The texture intensity drops sharply upon *t*-HPS before a minimum at an equivalent strain of ~ 16. This is a direct consequence of the instability of the initial as-received texture under the shear deformation of *t*-HPS. After the minimum, the monotonic increase in the texture intensity demonstrates the steady and continuous intensifying of the new and emerging {$\stackrel{\mathrm{-}}{1}{\text{10}}$}  <110> texture.

## Discussion

### General evolution of microstructure and texture of 5N Al upon *t*-HPS

The evolution of the microstructural parameters upon SPD processing has been the focus of much research^15,43–46^ and it is well-known that a grain refinement saturation is usually achieved at suitable high strain levels^41,47–50^. However, currently there is no similar consensus on the texture evolution.

**Figure 7:**
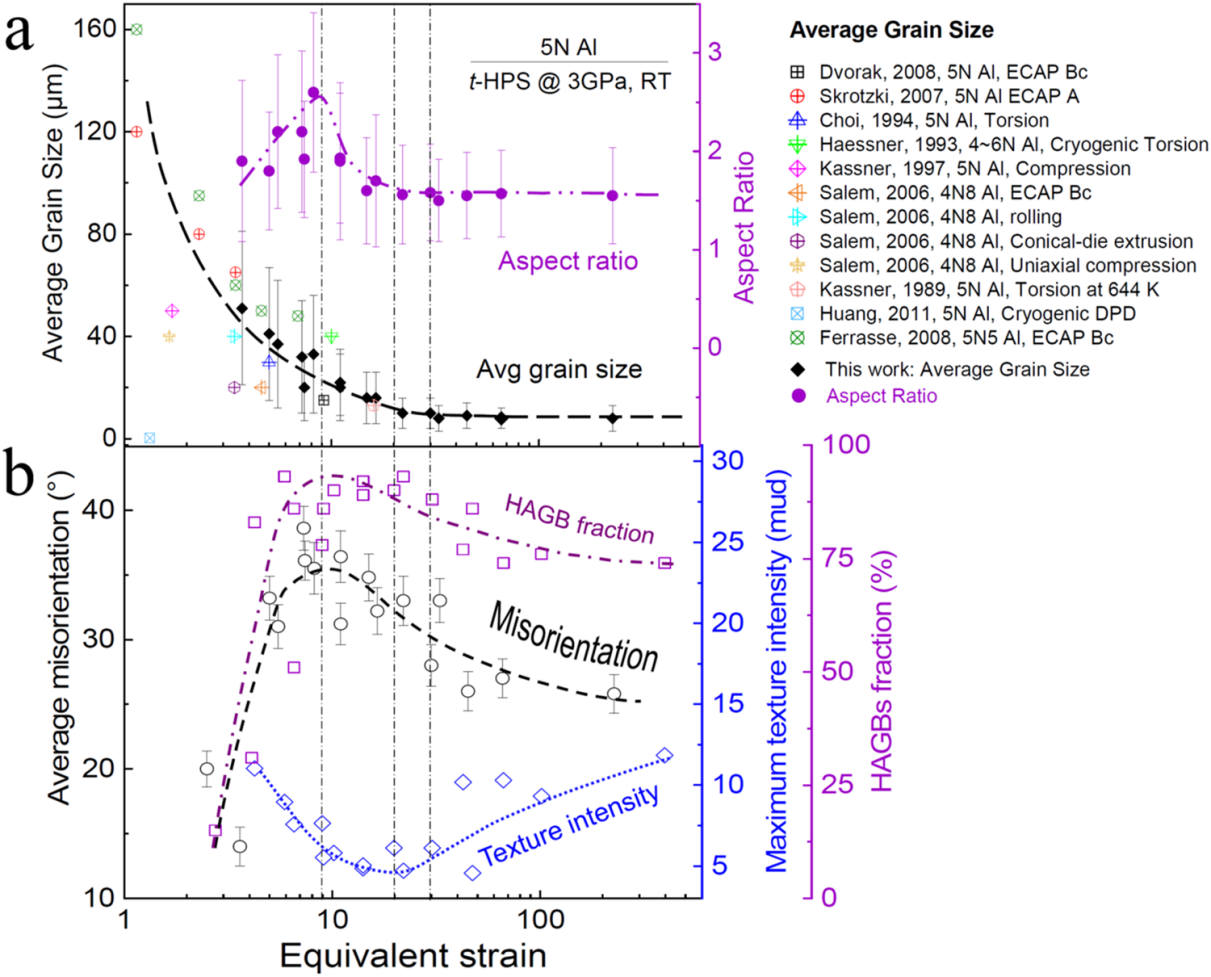
Evolution of microstructure parameters: (**a**) average grain sizes and grain aspect ratios; (**b**) average grain boundary misorientation, HAGB fractions and intensity of {$\stackrel{\mathrm{-}}{1}{\text{10}}$}  <110> texture obtained from (111) pole figure, upon increase of *t*-HPS equivalent strain: grain size data from the literature^36,51–58^ are also included.

Figure 7a summarizes the evolution of the morphological parameters, such as average grain size and aspect ratio, upon processing through equivalent strains taken from Figs. 4 and S1 where published grain size data^36,51–58^ were also included. It is readily apparent that the average grain size exhibits a strong correlation with the processing strain and the grain refinement saturates upon deformation processing at an equivalent strain of ~ 30. This fits well with published data and no additional significant grain refinement was achieved even when the processing strain was increased to more than ~ 200. It is already known from Fig. S1 that the grain size radial gradient diminishes to ~ 0 when the grain size reaches saturation because the saturation in refinement effectively eliminates the gradient. Furthermore, as indicated by Fig. 7a, the average aspect ratio of grains approaches a saturation, in parallel with the grain refinement, to a low value of ~ 1.6 at the same strain level of ~ 30. This is reasonable since numerous experiments have shown that the evolution of the microstructure and the properties during SPD processing tend towards homogeneity by straining^37,59–63^. Many metals processed by HPT begin to exhibit a saturation at equivalent strains between 10 and 30^41,64^.

Figures 5 and 7b show that the average grain boundary misorientations and HAGB fractions all evolve continuously with *t*-HPS strain, increasing initially at low strain levels accompanying the refinement of the grain structure, then passing through a peak at around an equivalent strain of ~ 9 and decreasing thereafter to monotonically approach saturation at high strain levels. These trends are shown clearly in Fig. 7b which summarizes the evolution of the average grain boundary misorientations, the HAGB fractions and the intensity of major textures obtained from the (100) pole figures in Figs. 5, 6 and S3. The intensity of the texture initially decreases sharply at low strain levels accompanying the grain refinement as the initial texture in the as-received undeformed sample is removed by the *t*-HPS shear, it passes through a minimum at around an equivalent strain of ~ 20 and increases monotonically thereafter as the new {$\stackrel{\mathrm{-}}{1}{\text{10}}$} < 110 > texture component gradually dominates. Again, and in parallel with the decreasing grain boundary average misorientation and HAGB fraction, the texture intensity tends to approach saturation but it is hard to estimate whether it is fully saturated even at processing equivalent strain levels exceeding ~ 200 where these are the largest strains attained in this investigation.

These results clearly demonstrate that, while morphological parameters such as the average grain size, grain size gradient and grain aspect ratio saturate at moderately high strain levels as in other SPD processes such as HPT, the HAGB fraction and most importantly the texture intensity evolve continuously and monotonically, even after the saturation of morphological parameters, and they tend to saturate at much higher strain levels in *t*-HPS.

By contrast, it was reported that the shear texture in torsion starts to form gradually at low shear strains and then weakens at higher shear strains^23^. An apparent texture fluctuation of the *B* and *C* components was also reported upon HPT straining and such oscillatory texture behavior around the ideal positions was confirmed in HPT simulations^65^.

It is generally believed that shear textures are rarely strong and there are no stable orientations in shear since any particular grain is constantly rotating so that the presence of a texture is simply the result of quasi-stationary positions in the orientation distributions where the grains rotate very slowly with respect to the specimen axes^23^. This apparent texture instability is also considered to be related to the complicated nature of torsional deformation^23^.

### Formation of a {$\stackrel{\mathrm{-}}{1}{\text{10}}$}  <110> texture

Figure 2b shows local orientation gradients in the coarse grain structure of the pressurized sample and Fig. 3 shows the similar microstructure in the unrecrystallized regions/grains in the sample processed by *t*-HPS to a rotation of *π*/6. These local orientation gradients can be related to the presence of Geometrically Necessary Dislocations. By rotating to *π*/4 and above, these unrecrystallized dislocation substructures disappear and are replaced by fully recrystallized microstructures as in Fig. 4. This microstructural transformation from a dislocation substructure to a recrystallized structure is accompanied by the loss of the initial (100) fiber texture and the emergence of a clearly identifiable {110} < $\stackrel{\mathrm{-}}{1}{\text{10}}$> texture at a *t*-HPS rotation of *π*/4. Thus, it is this new emerging {$\stackrel{\mathrm{-}}{1}{\text{10}}$}  <110> texture that ultimately replaces the initial as-received texture and the other possible deformation texture components developed at low strain levels, and thereafter it further develops into the sole component of a sharp texture accompanied by a fully recrystallized microstructure. This single component {$\stackrel{\mathrm{-}}{1}{\text{10}}$}  <110> texture is thus reasonably considered as a recrystallization texture.

**Figure 8:**
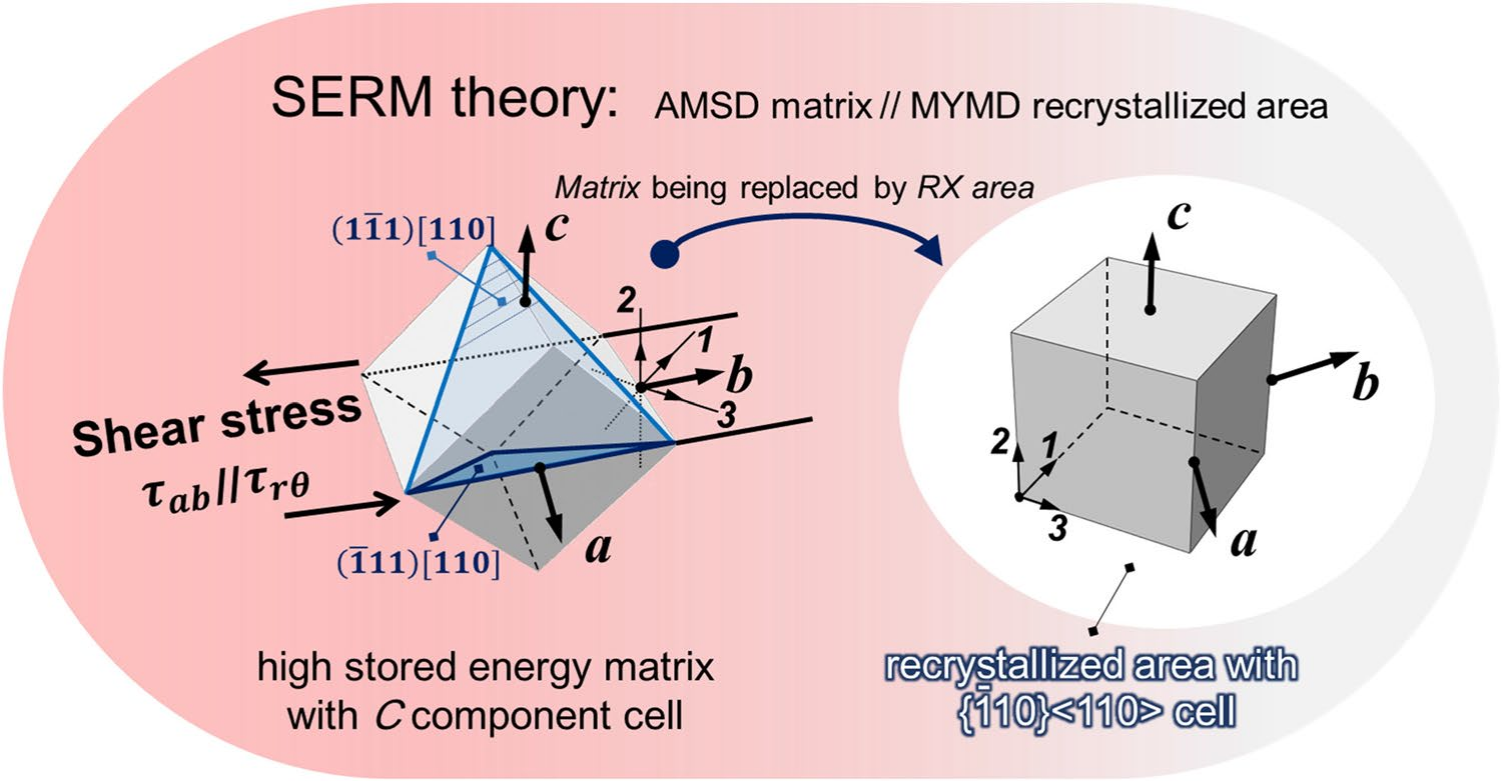
Illustration of the assumed *C* component 3D cubic cell in deformation state (left) and ideal {$\stackrel{\mathrm{-}}{1}{\text{10}}$}  <110> component 3D cubic cell in the recrystallized state (right); the sample local Cartesian coordinate system ***a***–***b***–***c*** and stress principal coordinate ***1–2–3*** are illustrated, and the local Cartesian coordinates ***a***, ***b*** and ***c*** are parallel to the sample cylindrical coordinates ***r,*** ***θ*** and ***z***, respectively.

Among the several models of recrystallization textures, the strain energy release maximization (SERM) theory^66^ appears to provide a possible explanation for the formation of the present single component texture. In general, the *C* component occupies a major part of the shear texture^67^. In Fig. 8 the orientation relationship between the *C* component unit cell and the sample local Cartesian coordinate system ***a***–***b***–***c***, as introduced in Fig. 1, is given in the deformed matrix and three principal directions of the local stress ***1–2–3*** are illustrated simultaneously. The only non-zero deviatory stress component *τ*_*rθ*_ in *t*-HPS^29^ leads to a dislocation stress field with directions consistent with *τ*_*rθ*_ in the *C*-oriented matrix.

The stress equation of the deviatory stress tensor $\sigma$ in the *C* component with local system ***a***–***b***–***c*** can be solved:

1 $${\sigma }^{3}-{J}_{1}\cdot {\sigma }^{2}-{J}_{2}\cdot \sigma -{J}_{3}=0$$

where *J*_1_, *J*_2_, and *J*_3_ are the invariants of the deviator stress tensor.

The three real roots of Eq. (1) are the three principal stresses whose directions cosines < *l m n* > in the local system ***a***–***b***–***c*** can be readily obtained by the following equations:

2 $$\left.\begin{array}{c}\left({\sigma }_{aa}-\sigma \right)\cdot l+{\tau }_{ba}\cdot m+{\tau }_{ca}\cdot n=0\\ {\tau }_{ab}\cdot l+\left({\sigma }_{bb}-\sigma \right)\cdot m+{\tau }_{cb}\cdot n=0\\ {\tau }_{ac}\cdot l+{\tau }_{bc}\cdot m+\left({\sigma }_{cc}-\sigma \right)\cdot n=0\\ {l}^{2}+{m}^{2}+{n}^{2}=1\end{array}\right\}$$

Since the local statistical resultant dislocation stress field will have only one shear component ${\tau }_{ab}$, thus ${\sigma }_{aa}={\sigma }_{bb}={\sigma }_{cc}=0 , {\tau }_{ac}={\tau }_{bc}=0,$ *J*_1_ = 0, *J*_2_ = ${\tau }_{ab}^{2}$, *J*_3_ = 0 for Eqs. (1) and (2) in the present case. The three sets of solutions are:

$$\begin{array}{ccc}{l}_{1}=-\frac{1}{\sqrt{2}}& {m}_{1}=\frac{1}{\sqrt{2}}& {n}_{1}=0\\ {l}_{2}=\frac{1}{\sqrt{2}}& {m}_{2}=\frac{1}{\sqrt{2}}& {n}_{2}=0\\ {l}_{3}=0& {m}_{3}=0& {n}_{3}=1\end{array}$$

According to the SERM theory, the absolute maximum stress direction (AMSD) (i.e. <100> for aluminum) of the recrystallized 3D unit cell will be parallel to the three principal stress directions and the three sets of < *l m n* > . The Miller index of the local sample coordinates ***a*** = [*a*_1_ *a*_2_ *a*_3_], ***b*** = [*b*_1_ *b*_2_ *b*_3_], ***c*** = [*c*_*1*_* c*_*2*_* c*_*3*_] expressed in the recrystallized 3D unit cell coordinates are then:

3 $$\left[\begin{array}{ccc}{a}_{1}& {a}_{2}& {a}_{3}\\ {b}_{1}& {b}_{2}& {b}_{3}\\ {c}_{1}& {c}_{2}& {c}_{3}\end{array}\right]\left[\begin{array}{ccc}{l}_{1}& {m}_{1}& {n}_{1}\\ {l}_{2}& {m}_{2}& {n}_{2}\\ {l}_{3}& {m}_{3}& {n}_{3}\end{array}\right]=\left[\begin{array}{ccc}1& 0& 0\\ 0& 1& 0\\ 0& 0& 1\end{array}\right]\left[\begin{array}{ccc}\frac{-1}{\sqrt{2}}& \frac{1}{\sqrt{2}}& 0\\ \frac{1}{\sqrt{2}}& \frac{1}{\sqrt{2}}& 0\\ 0& 0& 1\end{array}\right]=\left[\begin{array}{ccc}\frac{-1}{\sqrt{2}}& \frac{1}{\sqrt{2}}& 0\\ \frac{1}{\sqrt{2}}& \frac{1}{\sqrt{2}}& 0\\ 0& 0& 1\end{array}\right]$$

**Figure 9:**
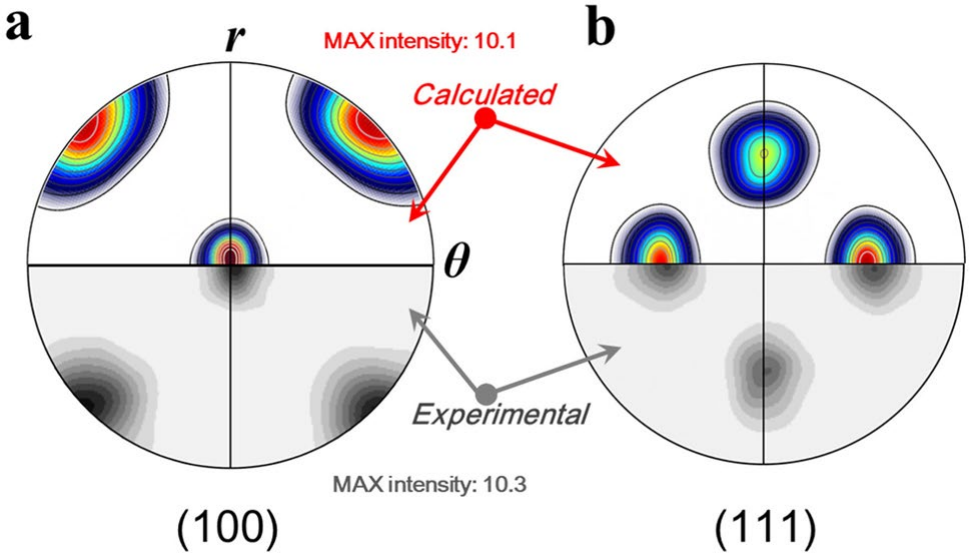
The (**a**) (100) and (**b**) (111) pole figures observed in 5N Al after *t*-HPS rotation to 2*π* (bottom), by comparison to those estimated (top) for the {$\stackrel{\mathrm{-}}{1}{\text{10}}$} <110> texture pole figure with a spreading of 27°.

This leads to ${\langle \frac{-1}{\sqrt{2}}\frac{1}{\sqrt{2}}0\rangle }_{R}$//${\langle \overline{1}10\rangle }_{R}$//***a***//***r***, ${\langle \frac{1}{\sqrt{2}}\frac{1}{\sqrt{2}}0\rangle }_{R}$//${\langle 110\rangle }_{R}$//***b***//***θ***, and ${\langle 001\rangle }_{R}$//***c***//***z***, where the subscript *R* denotes the lattice unit cell in the recrystallized area. This result is in full agreement with the ideal orientation determined by the experimental pole figure in Fig. 6, as is shown schematically in the recrystallized area of Fig. 8. Pole figures based on the SERM results can be created by calculating the characteristic Gaussian intensity distribution with a spreading angle of 27°. This spreading angle is chosen since the average misorientation is 27° ± 1.5°at *t*-HPS rotation of 2π, as shown in Fig. 5e. The calculated (100) and (111) pole figures were compared with that of the experimental pole figure from the sample with a *t*-HPS rotation angle of 2π as shown in Fig. 9. Thus, both qualitative patterns and quantitative intensities are fully consistent.

Therefore, the single-component texture of {$\stackrel{\mathrm{-}}{1}{\text{10}}$} <110> observed experimentally in this study may be formed directly from the *C*-oriented deformation texture.

### The stability of the {$\stackrel{\mathrm{-}}{1}{\text{10}}$}  <110> orientation during *t*-HPS

It is important to examine the stability of this sole component texture {$\stackrel{\mathrm{-}}{1}{\text{10}}$} <110> with ever increasing intensity as it is observed to be shear stable during *t*-HPS of the 5N Al at room temperature. In fact, this orientation was considered earlier to meet all the requirements of the preferred orientation for shear deformation, together with {001} <110> the well-known *C* component, for fcc metals^13,68^. Unfortunately, there was very little mention of this orientation^13,14^ and the experimental absence of this shear component was attributed directly to stability considerations^13^. It is also interesting to note that, according to SERM theory, a supposed shear texture of {$\stackrel{\mathrm{-}}{1}{\text{10}}$} <110> could also generate itself in recrystallization.

This has two important consequences. First, if the {$\stackrel{\mathrm{-}}{1}{\text{10}}$} <110> grains or substructures were indeed formed during *t*-HPS, even if in only a small fraction, they will survive in the present experimental situation since they are energetically favored to become recrystallization nuclei according to the SERM theory. These {$\stackrel{\mathrm{-}}{1}{\text{10}}$} <110> nuclei have large misorientations with all the known ideal shear components: 35.3° with *A* and $\stackrel{\mathrm{-}}{\text{A}}$, 46° with *A*_1_^*^ and *A*2*, 54.7° with *B*, or $\stackrel{\mathrm{-}}{\text{B}}$ and 62.8° with *C*. Hence, they will form high-mobility HAGBs having misorientations larger than 35° between the {$\stackrel{\mathrm{-}}{1}{\text{10}}$} <110> nuclei and the as-deformed matrix of other shear texture components. There is extensive evidence that HAGBs have a greater mobility than LAGBs^35^ and the high mobility of these HAGBs may play a decisive role in the orientation growth^69–72^ of the {$\stackrel{\mathrm{-}}{1}{\text{10}}$} <110> nuclei.

Second, since the {$\stackrel{\mathrm{-}}{1}{\text{10}}$} <110> texture component emerges during *t*-HPS, it maintains stability during subsequent processing when the monotonic deformation path is maintained. The subsequent recrystallization cycle continuously occurs on the previously recrystallized matrix with deformation and thereby produces the {$\stackrel{\mathrm{-}}{1}{\text{10}}$} <110> component according to the SERM theory. In fact, this will produce an ever strengthening {$\stackrel{\mathrm{-}}{1}{\text{10}}$} <110> and this is observed experimentally in the present investigation. Thus, the {$\stackrel{\mathrm{-}}{1}{\text{10}}$} <110> component is first identified in the sample with *t*-HPS rotation to *π*/4 (Fig. 6) where recrystallization is considered to be completed based on the clean grains bounded by HAGBs in Fig. 4 and the high HAGB fraction shown in Fig. 5b. The component then becomes the sole texture component with *t*-HPS rotation to *π* (Fig. 7) coupled with a fully recrystallized microstructure and the intensity then increases continuously upon further *t*-HPS straining. This means that the {$\stackrel{\mathrm{-}}{1}{\text{10}}$} <110> texture of the 5 N Al is stable during *t*-HPS at ambient temperature.

Room temperature rolling to 30% reduction (an equivalent true strain of ~ 0.42) of a cube orientated high-purity aluminum single crystal, leads to a normal distribution of crystal orientations within an angular limit of ± 3° around the ideal orientation of the original cube^73^. It is reasonable to speculate that the same order of grain orientation scattering may occur in *t*-HPS to the same equivalent strain. Such a scattering of the grain orientations is much smaller compared to the average grain boundary misorientation dictated in Fig. 5b after the {$\stackrel{\mathrm{-}}{1}{\text{10}}$} <110> texture becomes the sole texture during *t*-HPS processing. Also, such scattering may be readily rectified towards the ideal {$\stackrel{\mathrm{-}}{1}{\text{10}}$} <110> orientation by the subsequent cycle of recrystallization.

It is worth noting that an “oblique” cube component was reported earlier in 5N, 1050 aluminum and 4N Nickel in shear-dominated deformation of ECAP^52^, Friction-Assisted Lateral Extrusion^74^ and HPT^75^, which is the closest reported texture component to the present observed single component {$\stackrel{\mathrm{-}}{1}{\text{10}}$} <110> sharp texture.

The significant difference between the present investigation and previously reported “oblique” or rotated cube components^52,74,75^ relates to their stability. During ECAP of 5N Al in route A, after the 1st, 2nd and 3rd pass the component rotates anticlockwise around the transverse direction (equivalent to the z-axis of *t*-HPS) through 16°, 18° and 21°^52^. During HPT of 4N Ni, the component rotates towards a more stable *C* component during deformation at 523 K^75^. However, in the present work the {$\stackrel{\mathrm{-}}{1}{\text{10}}$} <110> component was formed and thereafter there was no rotation of this orientation and it was stable upon subsequent shear processing with continuous intensification. This is in distinct contrast to the stability feature of the “oblique” or rotated cube components observed earlier where the cube components are subjected to the orientation flow and rotate with the imposed rigid body spin under simple shear so that this component is considered unstable in simple shear^52,74,75^.

The stability of the {$\stackrel{\mathrm{-}}{1}{\text{10}}$} <110> orientation during *t*-HPS is examined above from the view point of microscopic evolution of the sample itself. This stability is also inevitably affected by the macroscopic stability of the process. One essential distinctive feature of the present *t*-HPS processing compared with conventional torsion or HPT is that the shear plane in HPT is the cross-sectional plane ***r–θ ***perpendicular to the sample axis whereas in *t*-HPS it is the cylindrical surface ***θ–z*** parallel to the sample axis^29,33^. The strain gradient along on the shear plane has thus a maximum along the radius and a value proportional to the radius in torsion and 0 in *t*-HPS. Therefore, *t*-HPS is considered closer to the ideal simple shear than torsion. Nevertheless, the physical significance arising from this difference remains to be investigated.

In a recent comprehensive analysis of texture evolution in HPT processing^22^, a link between the texture instability and the complicated nature of the torsional deformation was noted. By comparison, the present analysis provides initial explorations which demonstrate significant advantages of *t*-HPS over HPT or torsion in maintaining stable texture evolution.

## Conclusions

This investigation provides the first comprehensive report on the microstructural evolution of high purity aluminum (5N Al or 99.999% Al) processed by *t*-HPS up to a rotation of 10*π*. The results lead to the following significant conclusions:

1. The average grain size has a strong correlation with the processing strain. Grain refinement upon deformation processing reaches a saturation value of ~ 8 μm at an equivalent strain of ~ 30. No further significant grain refinement is achieved even when the processing strain is increased to more than ~ 200. The average aspect ratio of the grains evolves in parallel with the refinement in grain size and reach a low saturation value of ~ 1.6 at the same strain level of ~ 30. These trends of microstructural evolution fit well with previous reports obtained by SPD processing.
2. The average grain boundary misorientations and the HAGB fractions evolve continuously with the *t*-HPS strain, increasing first at low strain levels accompanying the refinement of grain structure, passing through a peak at an equivalent strain of about 8–9, and then decreasing monotonically thereafter and tending to saturate at high strain levels. This is in marked contrast to the evolution of the average grain boundary misorientation and the HAGB fraction observed in other SPD processes such as HPT where there is generally a saturation to a certain high strain level after a monotonic increase.
3. A {$\stackrel{\mathrm{-}}{1}{\text{10}}$} <110> texture emerges at a *t*-HPS equivalent strain of ~ 6 to 9, corresponding to a rotation of *π*/4, and it develops into a sole component strong texture which is accompanied by a fully recrystallized microstructure with ever increasing intensity upon *t*-HPS shearing. It tends to saturate when the processing equivalent strain reaches a level of ~ 200. This contrasts strongly with the rare achievement of a strong texture in torsion.
4. This strong single component {$\stackrel{\mathrm{-}}{1}{\text{10}}$} <110> texture is formed from the {001} <110> (*C* component) shear texture through recrystallization according to the Strain Energy Release Maximization (SERM) theory. Nevertheless, the possibility of an origin from the {$\stackrel{\mathrm{-}}{1}{\text{10}}$} <110> shear texture via recrystallization cannot be fully excluded.
5. The {$\stackrel{\mathrm{-}}{1}{\text{10}}$} <110> orientation is stable during *t*-HPS of 5N Al at room temperature where recrystallization becomes complete at strain levels above ~ 6. This orientation was not previously observed experimentally as a shear texture and its absence has been generally attributed to stability considerations.

## Materials and methods

Extruded aluminum rods with a purity of 99.999 wt% (5N Al) were coaxially machined into tubes with an inner radius of 12 mm, outer radius of 13 mm and a height of 15 mm, and then annealed for 2 h at 573 K and water-quenched. The tubular samples were processed by *t*-HPS to different rotation angles at ambient temperature with a constant shear direction along the azimuthal *θ* in cylindrical coordinates and with a constant hydrostatic pressure of ~ 3 GPa within the tube. The rotation angular velocity during *t*-HPS processing was *π*/100 per second. It is important to note that the sample coordinate system for *t*-HPS is different from that of conventional torsion in that, whereas the Shear Plane Normal (SPN) is the ***z***-axis of a torsion sample, the Shear Plane Normal (SPN) is the radius ***r***-axis of a *t*-HPS tube in this work^29,33,34^. Considering the intrinsic radial gradient of strain in *t*-HPS^29,33,34,76^, microstructural characterizations were carried out at three radial positions near the inner, middle and outer of the tube wall, respectively. Based on earlier reports^29,33^, the equivalent strains in the 5 N Al tube at different positions (inner, middle and outer) after *t*-HPS rotation to different angles (*π*/6, *π*/4, *π*/2, *π*, 2*π* and 10*π*) are listed in Table 1. An equivalent strain rate of 0.15–0.33 s^−1^ was estimated from the outer surface to the inner surface of the tube wall.

**Table 1:** The equivalent strain after *t*-HPS (rotation angle π/6 ~ 10π) at different observation regions.

Rotation angle	Regions			
	Inner	Middle	Outer	Average
*π*/6	5.5	3.6	2.5	3.8
*π*/4	8.2	5.5	3.7	5.7
*π*/2	16.5	11	7.4	11.3
*π*	33	22	15	22.7
2*π*	66	45	30	45.3
10*π*	330	227	148	227

The microstructure was examined using the electron backscatter diffraction (EBSD) technique by Oxford Instruments Nordlys Detector equipped on the HITACHI SU1510 Scanning Electron Microscope (SEM) operating at 20 kV. Data were post-processed by HKL acquisition Channel 5 software and JTEX non-commercial software. The sample preparation for EBSD was carried out by first grinding on 400-mesh to 2000-mesh waterproof abrasive paper followed by alumina solutions of 0.8 μm and 0.25 μm. Then electropolishing to a mirror-like surface was accomplished using an electrolyte of 15% perchloric acid and 85% ethanol at 253 K and a DC voltage of 15 V. The EBSD processing was performed with a step size from 5 to 0.05 μm depending on the studied areas and samples. The distributions of misorientations between neighboring grains were obtained based on the definition of a grain boundary by commercial orientation mapping software code Channel 5 from HKL Technology using an imposed condition of minimum misorientation between neighboring pixels. A boundary is defined where the threshold misorientation between two neighboring pixels is larger than 2° and an HAGB is when the misorientation is larger than 15°. An orientation step of 2° was used for misorientation frequency analyses.

## Supplementary Information

Supplementary Information.

## Data Availability

The materials or raw and processed data generated during this study will be made available from the corresponding author upon reasonable request.
