# Supplementary material for: Evidence for a stable single component sharp texture in high purity aluminum during tube high-pressure shearing at room temperature
Source: Sci Rep. 2022 Oct 25;12:17901. doi: 10.1038/s41598-022-21717-z (PMC9596461; doi:10.1038/s41598-022-21717-z)
Supplement: Supplementary file 1 — Supplementary Information. [file 41598_2022_21717_MOESM1_ESM.pdf]

Evidence for a stable single component sharp texture in high purity aluminum during tube high-pressure shearing at room temperature

Zheng Li[a], Luo Yi Li[a], Ye Bin Zhu[a], Kui Lin[a], Zhi Tian Ren[a], Yang Yang[a], Ying Liu [a],

Jing Tao Wang[a,*], Terence G. Langdon[b]

[a] School of Materials Science and Engineering, Nanjing University of Science & Technology, Nanjing 210014, China

[b] Materials Research Group, Department of Mechanical Engineering, University of Southampton, Southampton SO17 1BJ, U.K.

[*] Corresponding author, Jing Tao Wang, jtwang@njust.edu.cn

Supplementary material：Additional figures and tables

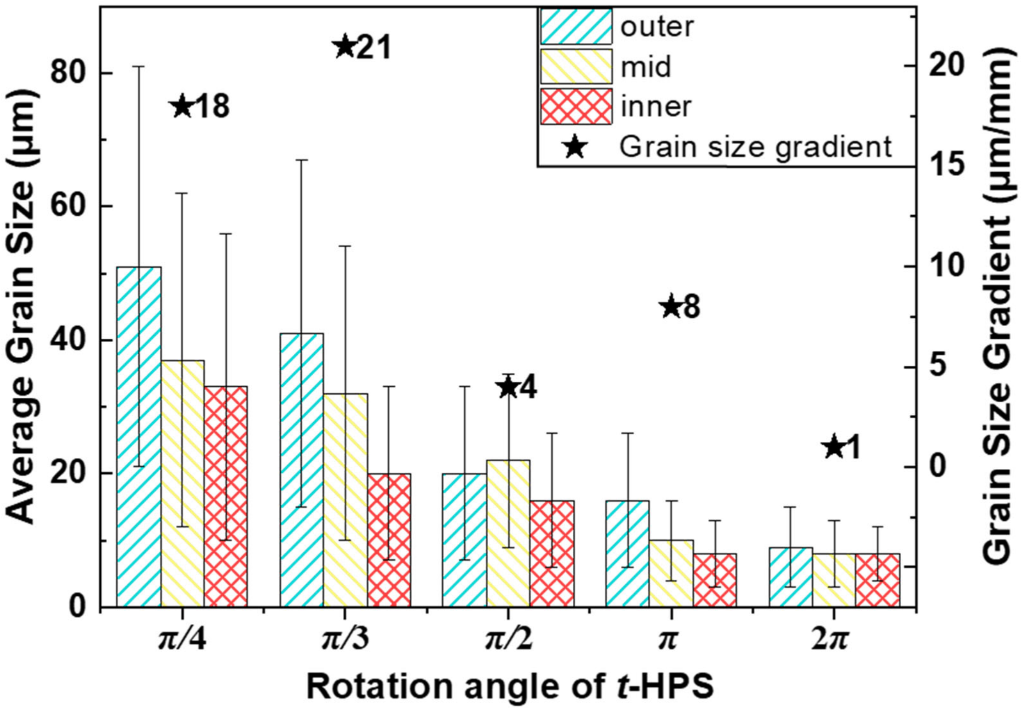

Figure S1. Evolution of average grains (columns) and grain size radical gradient (stars★) upon increase of *t*-HPS rotation angle.

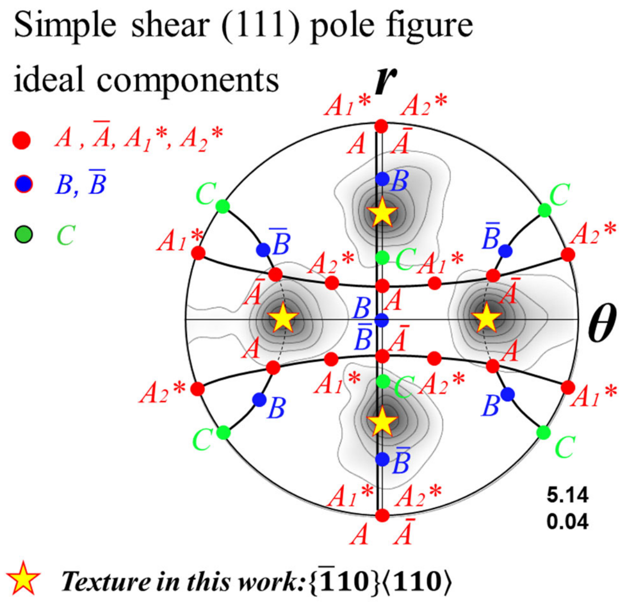

Figure S2. The (111) pole figure of {1̄10}<110> texture with relative intensity maximum of 5.14 of 5N Al obtained after *t*-HPS rotation to 2*π*, superimposed on (111) pole figure of reported ideal components under simple shear [1-4]. The ideal {1̄10}<110> location is expressed by red lineated yellow star.

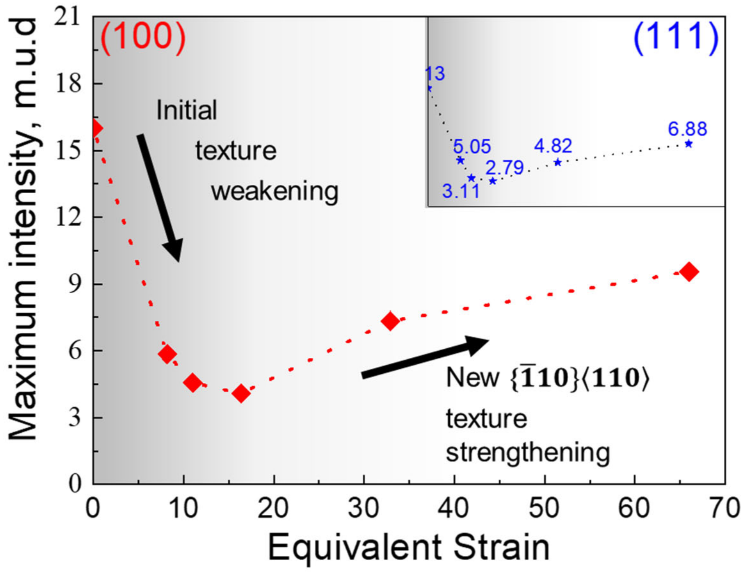

Figure S3. Evolution texture intensity obtained from (100) pole figure (red square) and (111) pole figure (blue star in the insert), upon increase of *t*-HPS equivalent strain.

Table S1. Main ideal texture components in simple shear deformation of fcc materials [1]

Notation	Miller indices	Euler angles(°) [a]			Fiber it belongs to
	{*hkl*}<*uvw*>	φ_1	Φ	φ_2	
A_1^*	$(111)[\bar{1}\bar{1}2]$	35.26/215.26	45	0/90	{111}-fiber
		125.26	90	45	
A_2^*	$(111)[11\bar{2}]$	144.74	45	0/90	{111}-fiber
		54.74/234.74	90	45	
A	$(1\bar{1}1)[110]$	0	35.26	45	{111}-fiber
$\bar{A}$	$(\bar{1}1\bar{1})[\bar{1}\bar{1}0]$	180	35.26	45	{111}-fiber
B	$(1\bar{1}2)[110]$	0/120/240	54.74	45	<110>-fiber
$\bar{B}$	$(\bar{1}1\bar{2})[\bar{1}\bar{1}0]$	60/180	54.74	45	<110>-fiber
C	{001}<110>	90/270	45	0/90	<110>-fiber
		0/180	90	45	

[a] Given in the $\varphi 2$ =0°, 45° and 90° sections with $\varphi 1$ = 0~270° only.

References

1. Li, S., Beyerlein, I. J. & Bourke, M. A. M. Texture formation during equal channel angular extrusion of fcc and bcc materials: comparison with simple shear. *Mater. Sci. & Eng.: A*. **394**, 66-77 (2005).
2. Montheillet, F., Cohen, M. & Jonas, J. J. Axial stresses and texture development during the torsion testing of Al, Cu and a-Fe. *Acta Metall.* **32**, 2077-2089 (1984).
3. Canova, G. R., Kocks, U. F. & Jonas, J. J. Theory of torsion texture development. *Acta Metall.* **32**, 211-226 (1984).
4. Montheillet, F., Gilormini, P. & Jonas, J. J. Relation between axial stresses and texture development during torsion testing: A simplified theory. *Acta Metall.* **33**, 705-717 (1985).
